# A Polymorphism in C-C Chemokine Receptor 5 (CCR5) Associates with Löfgren’s Syndrome and Alters Receptor Expression as well as Functional Response

**DOI:** 10.3390/cells10081967

**Published:** 2021-08-03

**Authors:** Bekir Karakaya, Coline H. M. van Moorsel, Marcel Veltkamp, Claudia Roodenburg-Benschop, Karin M. Kazemier, Annette H. M. van der Helm-van Mil, Tom W. J. Huizinga, Jan C. Grutters, Ger T. Rijkers

**Affiliations:** 1 Interstitial Lung Diseases Centre of Excellence, St. Antonius Hospital, P.O. Box 2500, 3430 EM Nieuwegein, The Netherlands; c.van.moorsel@antoniusziekenhuis.nl (C.H.M.v.M.); M.Veltkamp@antoniusziekenhuis.nl (M.V.); c.benschop@antoniusziekenhuis.nl (C.R.-B.); j.grutters@antoniusziekenhuis.nl (J.C.G.); 2 Division of Heart & Lungs, University Medical Center Utrecht, P.O. Box 85500, 3508 GA Utrecht, The Netherlands; k.m.kazemier@umcutrecht.nl; 3 Center for Translational Immunology, University Medical Center Utrecht, P.O. Box 85500, 3508 GA Utrecht, The Netherlands; 4 Department of Rheumatology, Leiden University Medical Center, P.O. Box 9600, 2300 RC Leiden, The Netherlands; a.h.m.van_der_helm@lumc.nl (A.H.M.v.d.H.-v.M.); t.w.j.huizinga@lumc.nl (T.W.J.H.); 5 Department of Science, University College Roosevelt, P.O. Box 94, 4330 AB Middelburg, The Netherlands; g.rijkers@ucr.nl

**Keywords:** sarcoidosis, Löfgren’s syndrome, C-C chemokine receptor 5 (CCR5), calcium influx, calcium kinetics, single nucleotide polymorphism (SNP)

## Abstract

C-C chemokine receptor 5 (CCR5) and polymorphisms in *CCR5* gene are associated with sarcoidosis and Löfgren’s syndrome. Löfgren’s syndrome is an acute and usually self-remitting phenotype of sarcoidosis. We investigated whether the single nucleotide polymorphism (SNP) rs1799987 is associated with susceptibility for Löfgren’s syndrome and has an effect on CCR5 expression on monocytes and function of CCR5. A total of 106 patients with Löfgren’s syndrome and 257 controls were genotyped for rs1799987. Expression of CCR5 on monocytes was measured by flowcytometry. We evaluated calcium influx kinetics following stimulation upon N-formylmethionyl-leucyl-phenylalanine (fMLP) and macrophage inflammatory protein-1α (MIP-1α) on monocytes by measuring the median fluorescence intensity (MFI). The frequency of the G allele of rs1799987 was significantly higher in Löfgren’s syndrome than in healthy controls (*p* = 0.0015, confidence interval (CI) 1.22–2.32, odds ratio (OR) 1.680). Patients with a GG genotype showed higher CCR5 expression on monocytes than patients with the AA genotype (*p* = 0.026). A significantly (*p* = 0.027) lower count of patients with the GG genotype showed a calcium influx reaction to simulation upon MIP-1 α, compared with patients with the AA genotype. The rs1799987 G allele in *CCR5* gene is associated with susceptibility to Löfgren’s syndrome and with quantitative and qualitative changes in CCR5, potentially effecting the inflammatory response.

## 1. Introduction

Sarcoidosis is a systemic inflammatory disorder of unknown cause with a wide clinical spectrum [1]. It commonly affects the lungs and intrathoracic lymph nodes, and is characterized by the formation of non-caseating epithelioid cell granulomas. During granuloma formation, there is a tight collaboration between macrophages, dendritic cells, and lymphocytes, orchestrated by cytokines and chemokines, which are potent chemoattractants for these cell types to sites of inflammation [2,3].

Chemokine receptors belong to the G-protein-coupled receptors (GPCR) superfamily and are divided into four classes, named by the type of chemokine (CC, CXC, CX_3_C, or C) with which they interact [4].

Signaling via G-protein-coupled receptors (GPCRs) is frequently linked to ion channels, which may induce temporary changes in cytoplasmic ion concentrations important in regulation of many functions of, for example, macrophages, such as innate host defense and secretory responses, including cytokine production. An important and frequently studied GPCR is C-C chemokine receptor 5 (CCR5), for which a role has been suggested in many different diseases like MS, HIV, and cancer, as well as autoimmune diseases like IBD, rheumatoid arthritis, and sarcoidosis [5,6,7,8,9,10].

Several polymorphisms have been described in the *CCR5* gene, of which rs1799987 at position -2459 (A > G) (also known as 303 A > G, 59029 A > G) promoter polymorphisms is of particular relevance. In human immunodeficiency virus (HIV)-infected patients, rs1799987 minor G allele is associated with slower progression of the disease [9].

In sarcoidosis, a haplotype (human haplotype C (HHC) ACTGTGC) of *CCR5* polymorphisms, including rs1799987 A > G (the underlined G in the Haplotype), was found to be associated with persistent lung involvement in both Dutch and British patients [11]. In a German study, two variants in the *CCR5* gene, other than rs1799987, which were also part of this HHC haplotype, were shown to be associated with Löfgren’s syndrome [12]. Löfgren’s syndrome is a self-limiting benign form of sarcoidosis, which presents with bilateral hilar lymphadenopathy, erythema nodosum (EN), and/or articular inflammation or arthritis [13]. However, in contrast with sarcoidosis, the disease is characterized by an acute onset, which can be invalidating for a limited period of time.

Apart from being a chemokine receptor, CCR5 also is a co-receptor, next to CD4, for HIV to enter the target cell [14]. Binding of HIV to CD4^+^ T cells promotes a chronic immune activation, which in turn upregulates CCR5 expression, creating a vicious circle driving HIV replication and progression of HIV infection. The rs1799987 G allele results in reduced expression of CCR5, thereby slowing the disease progression in HIV-infected persons [15].

We hypothesize that a decreased CCR5 expression contributes to a less intense inflammatory response and, therefore, a more benign course of disease as is present in patients with Löfgren’s syndrome. To better understand the role of rs1799987 in patients with Löfgren’s syndrome, we genotyped rs1799987 and measured the CCR5 expression on monocytes. Further, to explore if this polymorphism has functional consequences, we studied the kinetics and magnitude of changes in intracellular calcium of in vitro activated monocytes.

## 2. Materials and Methods

### 2.1. Subjects

A total of 106 unrelated Caucasian sarcoidosis patients, from two hospitals in the Netherlands (St. Antonius Hospital, Nieuwegein and Leiden University Medical Center, Leiden), were included in the study. All patients were diagnosed in accordance with the consensus of the ATS/ERS/WASOG Statement on sarcoidosiss [16]. All patients presented with the classic symptoms of Löfgren’s syndrome: acute onset with bilateral hilar lymphadenopathy, fever, erythema nodosum, and/or bilateral ankle arthritis.

Two hundred and fifty-seven healthy Caucasian subjects were included as controls in this study, matched by sex and ethnicity with the Löfgren’s syndrome patients. Written informed consent was obtained from all subjects, and authorization was given by the Ethics Committees of the St. Antonius Hospital, Nieuwegein and of Leiden University Medical Center. There was no significant difference in age or sex between Löfgren’s syndrome patients (mean age 34.8 years, 37.7% male) and controls (mean age 36.6 years, 35.4% male).

### 2.2. Genotyping

DNA was extracted from whole blood samples and the SNP analysis was performed using a custom GoldenGate Genotyping Assay (Illumina Inc, San Diego, CA, USA) performed in accordance with the manufacturer’s recommendations. We genotyped the *CCR5* polymorphism at position −2459 (promoter region, SNP rs1799987) for 106 patients and 257 controls.

### 2.3. Flow Cytometry

#### 2.3.1. CCR5 Expression on Peripheral Blood Monocytes

Cryopreserved PBMCs from 21 Löfgren’s syndrome patients were thawn and resuspended in phosphate-buffered saline (PBS). The cells were stained with CCR5-PECy7 (Ebioscience, San Diego, CA, USA), CD14-PerCP (monoclonal Peridinin-Chlorophyll-Protein, PerCP-labelled antibody, Becton Dickinson, San Jose, CA, USA), and CD16 PE (Phycoerythrin labeled antibody, Becton Dickinson). Mouse IgG1 kappa Isotype Control PE-Cy7 (Ebioscience) was used as negative control. The cells were measured on a FACS-Calibur (BD Biosciences, San Jose, CA, USA) and data analysis was performed using FlowJo software (v10.7, Ashland, OR, USA). Gating strategies for differentiation between classical, intermediate, and non-classical monocytes were performed as described before [17]. Monocytes were first gated according to their size and granularity characteristics in a FSC-SSC plot and then for CD14 expression. The percentage of CCR5 positive cells as well as the CCR5 median fluorescence intensity (MFI) expression levels were determined on CD14^+^ monocytes.

#### 2.3.2. Ca-Influx Assay

Sodium heparinized whole blood from the same 21 Löfgren’s syndrome patients, mentioned in paragraph 2.3.1, was lysed and cells were loaded with fluo4-AM (F14201, Invitrogen, Carlsbad, CA, USA), which was dissolved in DMSO (Sigma-Aldrich Co, St Louis, MO, USA) to a final concentration of 5 µM for 30 min. After centrifugation, the cells were stained with CD14-PerCP antibody (Becton Dickinson) and resuspended in 300 µL assay buffer composed of 1 mM CaCl_2_.2H_2_0, 5 mM Glucose-Hydrate, 5 mM KCl, 1 mM Na_2_HPSO_4_.2H_2_0, 0.5 mM MgSO_4_.7H_2_0, 145 mM NaCl, and 10 mM HEPES (pH 7.4). Cells then were stimulated with 3.8 pM MIP-1α (HPC1105, R&D systems, Minneapolis, MN, USA) or 5 nM fMLP (n-Formyl-Met-Leu-Phe F3506, Sigma-Aldrich Co, St. Louis, MO, USA) after baseline recording for 40 s and Ca-influx in time in CD14^+^ monocytes was recorded for a total of 200 s.

### 2.4. Quantification of Changes in [Ca^2+^]_i_ after Stimulation

**Figure 1:**
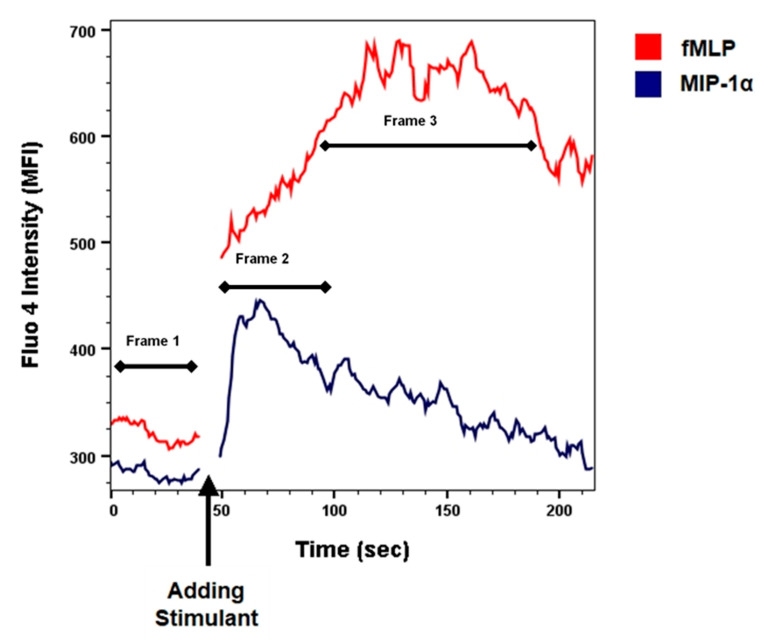
Flow cytometric analyses of kinetic changes in [Ca^2+^]_i_ in monocytes after stimulation with fMLP (5.0 nmol) or MIP-1α (3.8 pmol), in one representative patient. The arrow indicates the moment of adding the stimulant. The limiting factor in the monocyte calcium mobilization experiments was the number of cells available. For every patient (*n* = 21), two different stimuli were used: MIP-1α and fMLP. The number of cells available did not permit to perform replicates.

To quantify the changes of the [Ca^2+^]_i_, the median fluorescence intensity (MFI) of the fluo4 signal was measured after stimulation of the monocytes with fMLP or MIP-1α (macrophage inflammatory protein-1α). The monocytes were first gated on CD14 positivity followed by FSC-SSC scatter characteristics. In addition, because the variation in response to fMLP or MIP-1α differs in especially the start of the response to the stimuli in time and the duration of the [Ca^2+^]_i_ (Figure 1), we also used another approach to analyze the changes in [Ca^2+^]_i_. We measured the area under the curve and corrected this for the time (AUC/time) of the curve for three timeframes: 

Frame 1: Baseline, the [Ca^2+^]_i_ before stimulation with any chemoattractant (fMLP or MIP-1α). Mean time of 16.9 s (SD = 4.1 s).

Frame 2: MIP-1α, the increase in [Ca^2+^]_i_ upon stimulation MIP-1α. Mean time 21.0 seconds (SD = 2.8 s).

Frame 3: fMLP, the increase in [Ca^2+^]_i_ upon stimulation with fMLP. Mean time 49.1 seconds (SD = 10.9 s).

To see the net effect of stimulation with fMLP and MIP-1α, we subtracted the calcium influx baseline values from the values after stimulation with the ligand.

Formula (1).

a. MIP-1α (AUC/time)—Baseline MIP-1α (AUC/time) b. fMLP (AUC/time)—Baseline fMLP (AUC/time)(1)

We used fMLP as a positive control. The height of the Ca-influx after fMLP was different between the samples, so we also analyzed the difference in Ca-influx after MIP-1α compared with the possible maximum response after fMLP. To express the magnitude of the [Ca^2+^]_i_ response to MIP-1α relative to the fMLP response, and thereby to reduce inter-patient differences in the potency of the monocytes, we used the following formula:

Formula (2).

(2) $$\frac{(\mathrm{MIP}-1\alpha \;\left(\mathrm{AUC}/\mathrm{time}\right)—\mathrm{Baseline}\;\mathrm{MIP}-1\alpha \;\left(\mathrm{AUC}/\mathrm{time}\right)}{(\mathrm{fMLP}\left(\mathrm{AUC}/\mathrm{time}\right)—\mathrm{Baseline}\;\mathrm{fMLP}\left(\mathrm{AUC}/\mathrm{time}\right)}$$

### 2.5. Statistical Analysis

Allele and genotype frequencies were calculated for the SNP rs1799987 A > G polymorphism and tested for Hardy–Weinberg equilibrium (HWE) in controls. Differences between cases and controls were analyzed by χ^2^ test using contingency tables of genotype and allele frequencies. Hardy–Weinberg equilibrium (HWE), odds ratios, and confidence intervals (CIs) were calculated with an online tool, available at https://ihg.helmholtz-muenchen.de/ihg/snps.html (access on 2 August 2021). A *p*-value < 0.05 was considered significant.

To compare the mean percentage of CCR5 expression on monocytes, we performed one-way analysis of variance (ANOVA). The comparison of expression levels of CCR5 on monocytes among and between the genotypes was tested with the Kruskal–Wallis rank test and the Mann–Whitney-U rank test, respectively.

The occurrence of [Ca^2+^]_i_ in monocytes was tested with a χ^2^ test using a contingency table. The comparison of MFI of [Ca^2+^]_i_ in monocytes among and between the genotypes was tested with the Kruskal–Wallis rank test and respectively with the Mann–Whitney-U rank test. A *p*-value < 0.05 was considered significant. Statistical analyses were performed using the Statistical Program for the Social Sciences SPSS, version 26 (SPSS, Inc., Chicago, IL, USA).

## 3. Results

### 3.1. Genotyping

Patient and control groups were in Hardy–Weinberg equilibrium (*p* > 0.05). The frequency of the G allele of rs1799987 was significantly higher in Löfgren’s syndrome than in healthy controls (*p* = 0.0015, CI 1.22–2.32, OR 1.680) (Table 1). Furthermore, carriership of the G allele (GG + AG genotypes) was significantly increased in patients with Löfgren’s syndrome with 80% of Löfgren’s syndrome patients carrying the G allele versus 64% in controls (*p* = 0.0028; CI 1.31–3.88, OR 2.257).

### 3.2. CCR5 Expression on Peripheral Blood Monocytes

**Figure 2:**
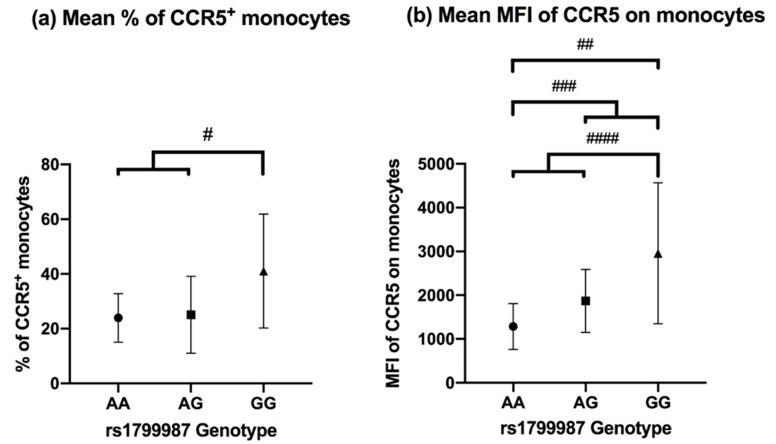
(**a**) Mean % of CCR5 positive (CCR5^+^) monocytes in 21 patients with the different genotypes for SNP rs1799987. Each patient was analyzed once. Comparison of AA vs. AG vs. GG (*p* = 0.094) was not significant, but comparison of GG vs. AA + AG showed a significant difference (*p* = 0.028) ^#^. (**b**) Mean MFI of CCR5 on monocytes in 21 patients with the different genotypes for rs1799987. Comparing AA vs. GG (*p* = 0.026) ^##^, AA vs. AG + GG (*p* = 0.038) ^###^, AA + AG vs. GG (*p* = 0.012) ^####^.

In patients with Löfgren’s syndrome, overall, 30.0% ± 16.6% of blood monocytes expressed CCR5. We investigated whether the percentage of monocytes expressing CCR5 was influenced by the presence of the G allele of rs1799987. There was no significant difference in the percentage of monocytes expressing CCR5 between the different genotypes (*p* = 0.094). However, a significantly higher percentage of CCR5^+^ monocytes was seen in patients with the GG genotype 41.06 (±20.80) versus AA + AG 24.53 (±11.32) (*p*-value = 0.028, Figure 2a).

We also analyzed the median fluorescence intensity (MFI) of CCR5 on the monocytes. There was a significant difference (*p* = 0.030) in MFI between the genotypes. Post-hoc analysis with pairwise comparisons showed for patients with the GG genotype significantly higher MFI compared with patients with the AA genotype (*p* = 0.026, after Bonferroni correction). Investigating the G allele showed similar results with patients having a higher MFI, patients with AG + GG versus AA genotypes showed significant higher MFI 2324.97 (±1382.38) versus 1283.65 (±523.47), *p* = 0.038 (Figure 2b). 

Further analysis of the monocyte subsets (classical, intermediate, and non-classical populations) did not show significant differences in CCR5 expression between the genotypes (data not shown).

CCR5 expression on lymphocytes was low and, in a number of cases, even undetectable (data not shown).

### 3.3. CCR5 Induced Calcium Mobilization Response in Monocytes

We performed the calcium mobilization assay in 21 patients. For the assay, we used the chemotactic peptide fMLP, which is known to induce changes in intracellular Ca ([Ca^2+^]_i_), as positive control.

Figure 1 illustrates the changes in [Ca^2+^]_i_ in monocytes after stimulation with fMLP and MIP-1α, a ligand for the CCR5 receptor.

In Figure 1, the first interval shows the baseline [Ca^2+^]_i_ level. After addition of fMLP and MIP-1α, a rise in [Ca^2+^]_i_ was observed in monocytes.

### 3.4. Kinetic Analysis of Changes in [Ca^2+^]_i_ after Stimulation

Stimulation of the monocytes with fMLP showed in all the 21 patients an immediate rise in [Ca^2+^]_i_, as illustrated by an elevated MFI, which persisted over the entire observation period of 200 s.

After stimulation of the monocytes with MIP-1α, 11 patients showed a rise in [Ca^2+^]_i_, similar to the figure presented earlier (Figure 1). Almost all patients who showed a rise in calcium influx upon stimulation with MIP-1α had the A allele (10 out of 11 patients) and mostly the AA genotype; this was significantly different from the patients who did not show any reaction, who mostly had the GG genotype (χ^2^ = 7.3, *p* = 0.027) (Table 2).

### 3.5. Quantification of Changes in [Ca^2+^]_i_ after Stimulation

The median fluorescence intensity (MFI) of the calcium influx, measured after stimulation of the monocytes with MIP-1α, did not show any significant difference between the different genotypes for the rs1799987. Measuring the difference in calcium influx after stimulation with MIP-1α with Formula (1) showed a lower [Ca^2+^]_i_ in patients with the GG genotype for rs1799987, but this was not significant, *p* = 0.11 (Table 3).

Calculating the magnitude of the [Ca^2+^]_i_ to MIP-1α relative to the fMLP response, applying Formula (2), the Kruskal–Wallis test showed a significant difference (*p* = 0.035) in [Ca^2+^]_i_ between the different genotypes. Post-hoc analysis showed a significantly lower [Ca^2+^]_i_ in patients with the GG genotype for rs1799987 (*p* = 0.042 after Bonferroni correction). Patients with the GG genotype compared with patients with the AA + AG genotype showed a significant lower [Ca^2+^]_i_, *p* = 0.010.

## 4. Discussion

In this study, we demonstrated that the G allele of SNP rs1799987 predisposes to Löfgren’s syndrome, influences CCR5 expression on monocytes, and decreases the functional response of the CCR5 receptor. Our data support our hypothesis that variation in CCR5 genetics and function contributes to a modified inflammatory response, which could explain the relatively benign course of sarcoidosis disease in patients with Löfgren’s syndrome.

Associations between polymorphisms of the *CCR5* gene and sarcoidosis were described earlier. Spagnolo et al. found an association between a specific haplotype (HHC), which includes rs1799987 A > G, and parenchymal involvement in patients with sarcoidosis. They did not find an association with susceptibility for sarcoidosis [11]; however they excluded patients with Löfgren’s syndrome. In a study with Löfgren’s syndrome patients from Germany, two marker alleles in the *CCR5* promoter region, other than rs1799987 A > G, but part of the HHC haplotype, were associated with Löfgren’s syndrome, in particular with female patients [12]. Interestingly, in patients with beryllium disease, which is a similar granulomatous disease as sarcoidosis, but with a known trigger, associations between worsening pulmonary function over time and *CCR5* gene polymorphisms were found. These gene polymorphisms were represented in the HHC haplotype [18].

Furthermore, associations between different inflammatory diseases and *CCR5* haplotypes or gene polymorphisms represented in the known *CCR5* haplotypes are described. *CCR5* haplotypes HHE and HHG*2 are associated with susceptibility to SLE [19]. Two *CCR5* gene polymorphisms (rs1799987 and rs10577983) are associated with radiographic severity of rheumatoid arthritis [20]. For the CCR5Δ32 deletion, an association with susceptibility and disease severity was established with primary sclerosing cholangitis. No association with the CCR5Δ32 deletion was found in patients with ulcerative colitis and Crohn’s disease [21].

In the present study, we chose to analyze only rs1799987 and no other SNP’s part of the *CCR5* haplotype, because the G allele of this SNP is part of haplotype HHC for which associations with sarcoidosis were found and, in HIV, the G allele was intensively analyzed, shown to slow HIV progression, independent of other polymorphisms, like the CCR5Δ32 deletion.

Löfgren’s syndrome is a characteristically Western and Northern European manifestation of sarcoidosis, more commonly seen in the Netherlands and Sweden [13]. According to gnomAD, the European population has a rs1799987 G allele frequency of 0.4327, which is similar to what we found in our cohort, and our cohort completely consists of Western Europeans.

In the present study, carriers of the G allele showed a higher expression of CCR5 on monocytes, which is in contrast to earlier reports, which showed increased CCR5 expression on the cell surface when carrying the A allele of rs1799987 [9,22]. The difference in cell types studied might be an explanation for the discrepancy in cell surface expression of CCR5. In the present study, CCR5 expression on monocytes was determined, while in previous reports, CCR5 expression on lymphocytes was addressed, where even T-cell subsets revealed different CCR5 expression [22,23,24]. It could be that this SNP does not affect the surface receptor expression on its own; for that, Shieh et al. [22] showed that there was no difference in surface expression of CCR5 on different cell types with the different genotypes of the rs1799987. However, individuals with the A allele for rs1799987 who also possessed the homozygous wild type of pCCR5-59653C showed a higher surface expression of CCR5 on different CD4^+^ cells. Another study showed that individuals with the rs1799987 A > G genotype showed lower CCR5 expression on stored peripheral blood CD4^+^ T cells and CD14^+^ monocytes, only when they were also heterozygous for the CCR5Δ32 deletion (Δ32/wt) [25].

Furthermore, the CCR5Δ32 deletion variant (Δ32/Δ32), which is not part of the HHC haplotype, results in a truncated protein that fails to reach the cell surface. However, variations in gene expression among Δ32/wt and wt/wt subjects have been described, suggesting other factors (e.g., other *CCR5* polymorphisms) contributing to CCR5 expression [26]. Altogether, data show that the effect of *CCR5* promoter polymorphisms on CCR5 expression may be cell type-specific and affected by other polymorphisms.

Another possible contributing aspect is the intracellular storage of CCR5 and its export to the plasma membrane after interacting with membrane associated or cytoplasmic proteins. In T-lymphocytes, the CCR5–CD4 interaction enhanced CCR5 transport to the plasma membrane [14].

In the present study, we showed that Löfgren’s syndrome patients with the G allele of the SNP rs1799987 had an impaired intracellular calcium influx, showing a dysfunctional chemokine–chemokine receptor interaction. One would not expect an effect of rs1799987 on the functionality of CCR5 given that this SNP is located in the promoter region. It could be that there are additional polymorphisms in the *CCR5* gene with strong linkage with rs1799987, which may have an impact on functionality, either in ligand binding and/or signaling function.

The expression and function of CCR5 is associated with differentiation of monocytes into macrophages as well as with phagocytosis and chemotaxis [27]. All these functions are crucial for regulation of inflammation and the formation and/or persistence of granuloma. We have shown previously that CCR5 is expressed at high levels on intermediate monocytes [17], suggestive for a role of this chemokine receptor in monocyte differentiation. We assessed the impact of rs1799987, but, maybe because of the low number of evaluable patients per group, did not find significant differences in the intermediate monocytes. 

Several studies [28,29,30] have shown a relation between chemokine signature and sarcoidosis. Higher CCR5 expression in BAL fluid (BALF) in sarcoidosis patients, regardless of sarcoidosis stage, and in Löfgren’s syndrome patients have been shown [31]. Significantly higher protein levels or mRNA expression of chemokine C-C motif chemokine ligand 5 (CCL5) were found in the BALF of sarcoidosis patients compared with controls [3,32]. Palchevsky et al. [29] showed in lung biopsies from sarcoidosis patients that different chemokines (CCL2, CCL5) and chemokine receptors (CCR2, CCR5) were found in different cell types creating the sarcoid lung granulomas, regardless of the radiologic stage of the sarcoidosis and whether or not alveolitis was present. Chemokines and chemokine receptors were reported to play a role in recruiting mononuclear cells that form and expand the granulomas during the earlier phases of pulmonary sarcoidosis [29]. 

After stimulation with chemoreceptors produced by APC (antigen presenting cell), CCR5 is recruited to the immunological synapse where it functions as a T cell costimulatory molecule by improving and prolonging the T cell–APC interaction [33]. A dysfunctional CCR5 could lead to a less stable T cell–APC interaction and, thereby, a shorter duration of the T cell–APC interaction, which in turn could lead to a less prolonged inflammatory state and an unstable granuloma formation in sarcoidosis.

The associations found between polymorphisms in CCR5, CCR2 [34], and sarcoidosis, including the present study, and the expression of the chemokines in the sarcoidosis granuloma suggest that genetic variants that cause decreased or dysfunctional chemokine receptors could lead to the formation of less stable granuloma, which in turn could lead to less prolonged disease, such as the Löfgren’s syndrome phenotype of sarcoidosis. It would be very interesting and important to replicate these findings in cells derived from patients with chronic sarcoidosis. Translating these findings to the clinical practice with potential treatment options should be a further interest of future studies. A case report about Maraviroc, which is a CCR5 inhibitor and used in HIV treatment [6], was recently published showing a resolution of sarcoidosis symptoms in an HIV-infected patient [35].

It is clear that CCR5 plays an important role in T cell function and that this depends on chemokines and cytokines in the environment at sites of infection and inflammation [36]. Chemokine receptors, like other GPCRs, function through calcium channels, which is of importance for the further functioning of the cell in the process [37].

For the calcium mobilization analyses, we used MIP-1α, a CCR5 ligand. The chemokine MIP-1α is a chemoattractant for CD8^+^ T cells, which also is shown to be produced by CD8^+^ T cell lymphocytes, and hence associated with a Th2 immune response [38,39].

Sarcoidosis patients with advanced stages (stage II and III) have a higher concentration of MIP-1α in BALF compared with controls [40]. Furthermore, a significant correlation between higher MIP-1α concentration and CD8^+^ T cell lymphocytes was observed in sarcoidosis patients with advanced stages of disease [40]. There seems to be a correlation between higher local MIP-1α concentrations and fibrotic lung changes, given the higher MIP-1α levels in progressive sarcoidosis and pulmonary fibrosis [41]. The expression of MIP-1α in interstitial fibroblasts found in biopsies obtained from patients with sarcoidosis and IPF emphasizes this [42]. In Japanese sarcoidosis patients, the plasma MIP-1α concentrations showed correlation with the course of disease, showing a decline in MIP-1α concentration in patients with spontaneous recovery [43]. In our group of patients with the G allele for rs1799987, the calcium mobilization response in monocytes following ligation of CCR5 with MIP-1α was impaired. This could be interpreted as dampening of otherwise inflammatory signaling towards Th2 dominated inflammation, or even directing the inflammation more towards a Th1-type inflammation and inducing a more adequate Th1 response, resulting in a benign course. This allele and, thereby, the dysfunctional CCR5 could be important in conducting the T cell inflammation.

The observations made in the present study may fit into a more complex mechanism contributing to the favorable prognosis of Löfgren’s syndrome. Löfgren’s syndrome is known for its good prognosis in about 90% of the patients, which is characterized by remission of the inflammation within 2 years, even without treatment with immunosuppressive drugs. 

Our data show that the G allele of SNP rs1799987 is overrepresented in patients with Löfgren’s syndrome, and that this allele associates with quantitative and qualitative changes in CCR5, potentially dampening the inflammatory response. Additional research is needed to further decipher the role of CCR5 expression and function in sarcoidosis before targeted treatment approaches may be considered.

## Figures and Tables

**Table 1:** Allele and genotype frequencies of rs1799987 in controls and patients with Löfgren’s syndrome.

CCR5 rs1799987	Controls (*n* = 257)	Löfgren’s Syndrome (*n* = 106) *
A (%)	299 (58%)	96 (45%)
G (%)	215 (42%)	116 (55%)
AA (%)	92 (36%)	21 (20%)
AG (%)	115 (45%)	54 (51%)
GG (%)	50 (19%)	31 (29%)

* Allele G was significantly increased in Löfgren’s syndrome patients (*p* = 0.0015, confidence interval (CI) 1.22–2.32, odds ratio (OR) 1.680), and there was a significant increase in the number of patients with Löfgren’s syndrome carrying the G allele (GG + AG genotypes; 80% in Löfgren’s syndrome patients versus 64% in controls *p* = 0.0028, CI 1.31–3.88, OR 2.257).

**Table 2:** [Ca^2+^]_i_ upon stimulation with MIP-1α in patients with Löfgren’s syndrome according to rs1799987 genotypes.

Stimulation with MIP-1α		
Genotype	Increase in [Ca^2+^]_i_ *	No Increase in [Ca^2+^]_i_
AA	6	1
AG	4	3
GG	1	6
Total	11	10

* Significantly more patients with the AA genotype showed a calcium influx reaction compared with patients with the GG genotype (χ^2^ = 7.3, *p* = 0.027).

**Table 3:** [Ca^2+^]_i_ measurements calculated upon stimulation with MIP-1α in patients with Löfgren’s syndrome according to rs1799987 genotypes.

Genotype	MIP-1α *	MIP-1α/fMLP **
AA	23.25	0.38
AG	16.35	0.24
GG	5.35	0.07

* Stimulation with MIP-1α, as calculated with Formula (1) in Material and Methods (M and M) to calculate the net effect of the stimulant. ** Stimulation with MIP-1α relative to fMLP, as calculated with Formula (2) in M and M to reduce inter-patient differences in monocyte potency.

## Data Availability

The data presented in this study are available on request from the corresponding author.
